# Anti-Programmed Death Ligand-1 Induced Acute Vision Loss in a Patient With Cancer-Associated Retinopathy

**DOI:** 10.7759/cureus.21709

**Published:** 2022-01-29

**Authors:** Muhammad Z Chauhan, Hana A Mansour, Maroof K Zafar, Sami H Uwaydat

**Affiliations:** 1 Department of Ophthalmology, University of Arkansas for Medical Sciences, Little Rock, USA; 2 Department of Ophthalmology, American University of Beirut, Beirut, LBN; 3 Department of Biochemistry, University of Arkansas for Medical Sciences, Little Rock, USA

**Keywords:** tulp1, paraneoplastic, vision loss, programmed death ligand-1, cancer-associated retinopathy

## Abstract

Cancer-associated retinopathy (CAR) is a potentially blinding disease triggered by autoimmunity to cancer antigens at distant sites. It may masquerade as immune-related adverse events from the use of immune checkpoint inhibitors (ICIs). We present a patient with an underlying tubby-related protein 1 (TULP1) cancer-associated retinopathy who lost vision following initiation of atezolizumab for small-cell lung cancer. This 75-year-old man presented with no light perception, paramacular and peripheral retinal pigmentary changes, attenuated outer retina, and extinguished rod and cone responses. The visual loss followed the induction of atezolizumab therapy. Possible atezolizumab-associated acute macular neuroretinopathy was considered, and atezolizumab was discontinued. Vision improved on oral corticosteroid and deteriorated when corticosteroid was tapered quickly. Retinal autoantibody serology testing was negative for both anti-recoverin and anti-enolase and was positive for anti-TULP1 autoantibodies. Re-induction of atezolizumab concomitant with high-dose oral and intravitreal corticosteroids resulted in visual recovery at the three-month follow-up. These findings suggest that ICI therapy for cancer can exacerbate the retinal dysfunction in a patient with underlying autoimmunity from cancer. Patients with a high risk of CAR may need to be evaluated for retinal autoantibodies before initiation of ICI.

## Introduction

Cancer-associated retinopathy (CAR) is a rare autoimmune paraneoplastic condition characterized by painless vision loss over weeks to months. Autoantibodies develop against tumor antigens that cross-react with proteins in the photoreceptors or retinal ganglion cells, ultimately leading to cell death and degeneration of the retina [1]. Symptoms depend on the retinal cell involved and can include photopsia, floaters, nyctalopia, peripheral visual field defect, and vision loss. The most common carcinomas associated with CAR arise from lung, colon, and ovarian/endometrial tissue [2]. Among numerous CAR autoantibodies, such as anti-recoverin, anti-α-enolase, anti-GAPDH (glyceraldehyde 3-phosphate dehydrogenase), anti-carbonic anhydrase II, and anti-aldolase [3], one autoantibody associated with endometrial cancer targets tubby-like protein 1 (TULP1) that supports the functional integrity of endocytic proteins in photoreceptors and is also detected in the retinal ganglion, Muller, and retinal pigment epithelial cells [4]. Mutations in TULP1 have been shown to cause retinal dystrophy [5]. Immune-related adverse events from the use of immune checkpoint inhibitors in cancer treatment include iritis, uveitis, scleritis [6]. Acute macular neuroretinopathy (AMN) has been reported in association with atezolizumab, which is a humanized monoclonal antibody that targets programmed death-ligand 1 (PD-L1) and 2 (PD-L2) for the treatment of triple-negative breast cancer and non-small cell lung cancer [7,8]. We report a patient with acute vision loss following treatment with atezolizumab for small lung cancer, who was subsequently diagnosed with CAR caused by autoantibodies against TULP1. Vision loss was reversible when oral and local steroids were administered.

## Case presentation

This 75-year-old man was referred for retinal evaluation following the sudden onset of bilateral vision loss, floaters, and photopsia. Past ocular history only included a corneal abrasion seven years prior. Past medical history included chronic obstructive pulmonary disease, bladder cancer, asbestos exposure, diabetes mellitus, and Barrett's esophagus. One month before the visual loss, the patient was diagnosed with small cell lung cancer when he presented with hoarseness and shortness of breath due to bulky mediastinal lymphadenopathy. Treatment with atezolizumab, carboplatin, and etoposide was started. Approximately three weeks after the first infusion of atezolizumab, he presented to an emergency department with complete loss of vision in his right eye. He had previously noted waxing and waning vision loss in the same eye with paracentral scotomas. A high-resolution magnetic resonance imaging of the brain did not reveal any acute pathology. The patient’s oncologist was aware of potential ocular side effects of atezolizumab and halted therapy. A presumed diagnosis of anti-PD-L1-associated acute macular neuroretinopathy (AMN) was considered.

**Figure 1:**
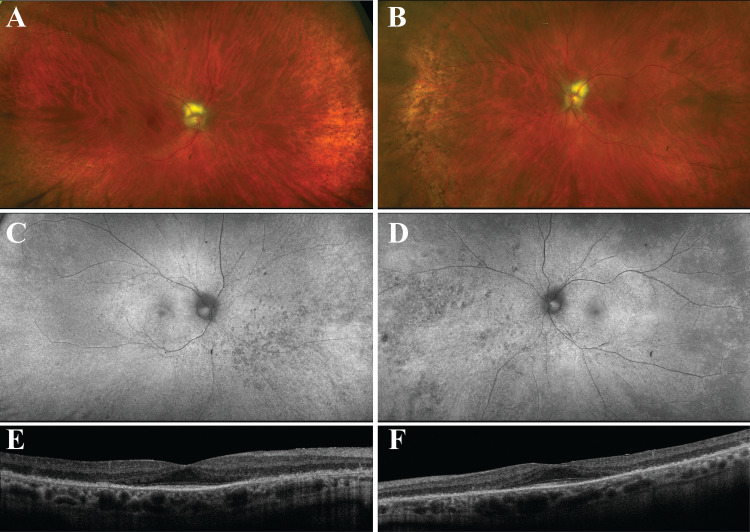
Multicolor wide-field photos, fundus autofluorescence (FAF), and optical coherence tomography (OCT) from patient with TULP1 cancer-associated retinopathy A, B: Multicolor wide-field photos showing fundus appearance of the right (A) and left (B) eye. Note attenuated retinal vessels and subtle pigmentary changes in the peripheral retina of both eyes. C, D: FAF of the right (C) and left (D) eye. Note hypo-FAF speckling bilaterally in the peripheral retina. E, F: Spectral domain OCT revealed loss of outer retinal layers outside the fovea in the right (E) and left (F) eye.

The patient was referred to the retina clinic one week after he visited the emergency department. At initial presentation, his visual acuity (VA) was no light perception (NLP) in both eyes with intraocular pressure (IOP) of 11 mm Hg in the right and 10 mm Hg in the left. Pupils were round and equal but poorly reactive to light. Mild nuclear sclerosis was noted bilaterally. Fundus exam revealed pink and sharp optic nerves with a wedge of myelinated nerve fiber layer, normal macula, attenuated retinal vessels, and subtle pigmentary changes in the peripheral retina of both eyes (Optos California system, Dunfermline, Scotland) (Figure 1A, 1B). Retinal imaging did not change through time. The fundus appearance was stable throughout the follow-up. Fundus autofluorescence (FAF) showed attenuated retinal vessels and a speckled hypo-FAF in the periphery (Figure 1C, 1D). Spectral-domain optical coherence tomography (OCT) (Zeiss Cirrus 5000, Dublin, California, USA) showed loss of outer retinal layers outside the fovea (Figure 1E, 1F).

An electroretinogram (ERG) (UTAS system, LKC Technologies, Gaithersburg, Maryland, USA) revealed extinguished rod and cone responses, with the residual cone responses having delayed amplitudes consistent with a profound retinal dysfunction. The patient’s presentation was not consistent with AMN. However, given the timeline of rapid onset vision loss after staring atezolizumab, the drug continued to be withheld. The patient was started on 80 mg prednisone for the presumed diagnosis of cancer-associated retinopathy (CAR). Three weeks later, there was a visual improvement to 20/30 in the right eye and 20/40 in the left eye. Pupils became reactive to light. The dose of oral prednisone was tapered.

One month later, the patient’s vision began to worsen when the dose of prednisone was tapered to 10 mg. Visual acuity dropped to hand motion in both eyes with sluggish pupillary response to light. Prednisone dosage was raised to 80 mg. However, at follow-up two months later, there was little to no improvement in VA. Fundus exam and OCT images were unchanged. A repeat ERG showed persistence of the extinguished rod and cone responses, with minimal improvement in cone function. An autoantibody test was ordered and revealed autoantibodies to TULP1 (Cancer-associated retinopathy panel by Immunoblot and Immunohistochemistry, Ocular Immunology Laboratory, Oregon Health & Science University, Portland, Oregon). Genetic testing was completed using the My Retina Tracker Program Panel Plus (version 4) (Blueprint Genetics, Seattle, Washington, USA). Genetic sequence analysis identified a heterozygous mutation in the nephrocystin-1 (NPHP1) gene, which was not expected to be related to the patient’s retinopathy. Administration of 4 mg intravitreal preservative-free triamcinolone acetonide (Triesence, Alcon, Fort Worth, Texas, USA) was given to the right eye, along with the high dose oral prednisone with a slow taper. Subsequently, the patient was started back on atezolizumab at the request of the oncologist, who also chose to initiate treatment with rituximab to control the autoimmune retinopathy and taper the prednisone. At the three-month follow-up, best-corrected visual acuity (BCVA) improved to 20/40 in the right eye and 20/30 in the left. Both fundus exam and OCT findings were stable at this time.

## Discussion

The patient detailed in this report presented with complete vision loss three weeks after starting atezolizumab immunomodulatory therapy for the treatment of small-cell lung cancer. The oncologist initially attributed the vision loss was due to anti-PD-L1-associated acute macular neuroretinopathy (AMN), and therapy was halted. However, ocular findings were not consistent with AMN. Discontinuation of atezolizumab along with initiating high-dose oral prednisone led to improvement in symptoms. There was a high index of suspicion for CAR upon disease progression with tapering of prednisone. Autoantibody panel revealed retinal antibodies to TULP1. Findings from this case are consistent with an atezolizumab exacerbated CAR.

**Figure 2:**
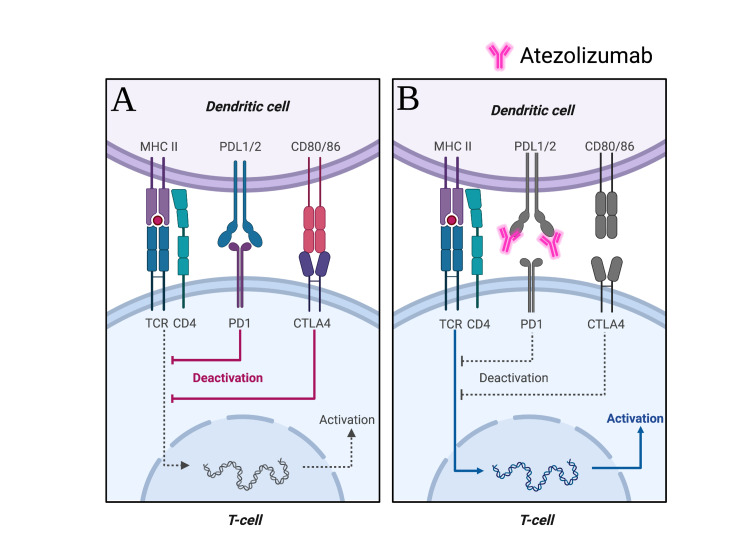
Role of atezolizumab in activation of the immune response A: The binding of the PD-L1 ligand inhibitory receptors to PD-1 on T cells deactivates T-cell activity. B: Atezolizumab is a humanized monoclonal antibody immune checkpoint inhibitor that binds to PD-L1, eliminates immune response inhibition, and breaks peripheral tolerance, potentially leading to immunological-mediated adverse effects. Created with BioRender.

Checkpoint blockade therapies, such as atezolizumab, nivolumab, and pembrolizumab, broadly work by negatively regulating inhibitory signals that mediate T-cell immunity. However, immune checkpoint inhibitors (ICIs) can influence B-cells. The two main immune checkpoint receptors with FDA-approved antibodies blocking them are cytotoxic T lymphocyte-associated antigen 4 (CTLA-4), PD-1, and its ligand PD-L1 [9]. Atezolizumab is a humanized monoclonal antibody that targets PD-L1 and PD-L2. The central function of PD-1 is to maintain peripheral tolerance through its interaction with PD-L1 (Figure 2A)* *[10]. Upon binding of PD-1 on T-cells to its ligand, there is downregulation of immune response. Thus, anti-PD-L1 drugs work by releasing this inhibitory signal (Figure 2B). The use of ICIs can disrupt immune homeostasis and may thereby lead to or exacerbate autoimmune diseases. For example, PD-1−/− knockout mice have been shown to develop lupus-like glomerulonephritis with deposition of renal IgG3 and C3 [11]. Immune checkpoint inhibitors have also been associated with ocular side effects [7,12,13]. Bitton et al. reported a prevalence of 0.4% of moderate-to-severe ocular complications following anti-PD-L1 treatment [14]. The clinical presentation of anti-PD-L1 associated retinopathy (AMN) is characterized by painless vision loss and paracentral scotoma with visual symptoms occurring around two weeks post-infusion that improve only partially after drug cessation. Clinical findings in our patient were not consistent with AMN.

**Figure 3:**
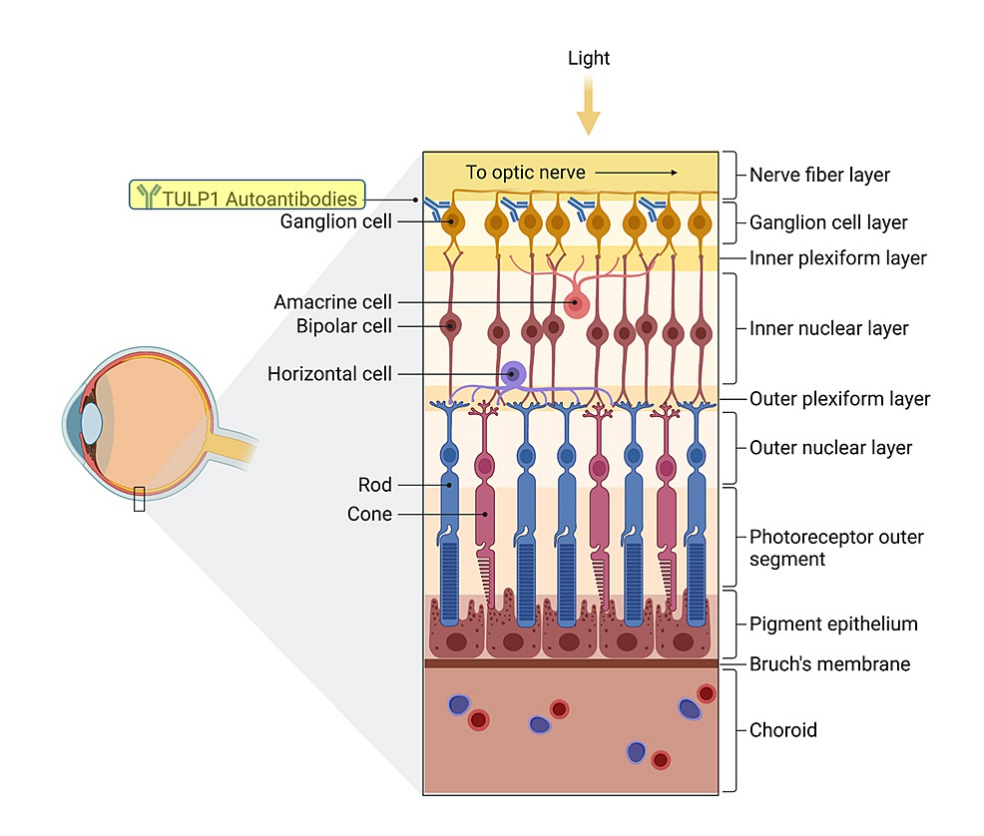
Generation of autoantibodies to TULP1 in retinal ganglion cells (RGCs) TULP1 has been identified in human RGCs and progenitor cells. Administration of atezolizumab in the patient detailed in this report with underlying TULP1 autoantibodies likely lead to disinhibition of autoimmunity, enhanced production of anti-TULP1 antibodies to RGCs, and subsequent rapid exacerbation of CAR. Created with BioRender.

In addition to leading to de novo autoimmune disease, anti-PD-L1 treatment may augment pre-existing autoimmune conditions. ICIs can cause a flare-up in an underlying autoimmune disease up to 50% of the time [15]. We believe that the patient detailed in this report had a CAR related to autoantibodies against TULP1 soon after he developed cancer and before starting atezolizumab but maintained good central vision. OCT showed loss of the outer retinal layers around the fovea, with a relatively preserved retinal architecture in the fovea, which explains the preserved central vision. TULP1 is a protein important for proper retinal cell function and survival and is expressed in photoreceptors and non-photoreceptor cells, such as retinal ganglion cells [16]. Initiation of atezolizumab in this patient with underlying TULP1 autoantibodies likely lead to disinhibition of autoimmunity, with enhanced production of anti-TULP1 antibodies, and subsequent rapid exacerbation of CAR with the severe visual loss [17]. The fact that TULP1 can be found on retinal ganglion cells may explain the acute worsening, then improvement of the patient’s vision from no light perception to 20/30 and 20/40 after treatment with steroids (Figure 3). Sustainability of visual improvement after re-starting atezolizumab coupled with administration of both intravitreal corticosteroid and resumption of systemic immunosuppression provides additional evidence that the autoantibodies worked as blockers in non-photoreceptor cells, with the blockage reversed when the antibodies were cleared.

## Conclusions

We present the case of a patient with underlying TULP1 cancer-associated retinopathy who lost vision following initiation of atezolizumab for small-cell lung cancer. With the increased use of immune checkpoint biologics, we recommend that before treatment with ICIs, especially anti-PD-L1 therapy, patients with a history of lung, colon, or ovarian cancer, who experience visual disturbances (new onset of floaters, night blindness, peripheral field defects, or vision loss) should be evaluated by an ophthalmologist for CAR. If autoantibodies are detected, immunosuppressive therapy should be initiated concomitantly with the ICI treatment aiming at a decrease in the incidence of immune-related vision loss.
